# Shedding the Light on the Natural History of Intracranial Aneurysms: An Updated Overview

**DOI:** 10.3390/medicina57080742

**Published:** 2021-07-22

**Authors:** Alice Giotta Lucifero, Matías Baldoncini, Nunzio Bruno, Renato Galzio, Juha Hernesniemi, Sabino Luzzi

**Affiliations:** 1 Neurosurgery Unit, Department of Clinical-Surgical, Diagnostic and Pediatric Sciences, University of Pavia, 27100 Pavia, Italy; alicelucifero@gmail.com; 2 Department of Neurological Surgery, Hospital San Fernando, Buenos Aires 1646, Argentina; drbaldoncinimatias@gmail.com; 3 Division of Neurosurgery, Azienda Ospedaliero Universitaria Consorziale Policlinico di Bari, 70124 Bari, Italy; bruno.nunzio@hotmail.it; 4 Neurosurgery Unit, Maria Cecilia Hospital, 48032 Cotignola, Italy; renato.galzio@gmail.com; 5 Juha Hernesniemi International Center for Neurosurgery, Henan Provincial People’s Hospital, Zhengzhou 450000, China; juha.hernesniemi@icloud.com; 6 Neurosurgery Unit, Department of Surgical Sciences, Fondazione IRCCS Policlinico San Matteo, 27100 Pavia, Italy

**Keywords:** endothelial disfunction, inflammatory cascade, intra-aneurysmal flow, intracranial aneurysm, subarachnoid hemorrhage, wall shear stress

## Abstract

The exact molecular pathways underlying the multifactorial natural history of intracranial aneurysms (IAs) are still largely unknown, to the point that their understanding represents an imperative challenge in neurovascular research. Wall shear stress (WSS) promotes the genesis of IAs through an endothelial dysfunction causing an inflammatory cascade, vessel remodeling, phenotypic switching of the smooth muscle cells, and myointimal hyperplasia. Aneurysm growth is supported by endothelial oxidative stress and inflammatory mediators, whereas low and high WSS determine the rupture in sidewall and endwall IAs, respectively. Angioarchitecture, age older than 60 years, female gender, hypertension, cigarette smoking, alcohol abuse, and hypercholesterolemia also contribute to growth and rupture. The improvements of aneurysm wall imaging techniques and the implementation of target therapies targeted against inflammatory cascade may contribute to significantly modify the natural history of IAs. This narrative review strives to summarize the recent advances in the comprehension of the mechanisms underlying the genesis, growth, and rupture of IAs.

## 1. Introduction

Intracranial aneurysms (IAs) are life-threatening cerebrovascular pathologies with an incidence and prevalence of 1–6% and 3.2%, respectively, in the adult population [1].

IAs are focal enlargements of the arterial wall owing to the destruction of internal elastic lamina and tunica media. The sudden rupture of these thin-wall regions causes a subarachnoid hemorrhage (SAH), the latter accounting for more than 25% of strokes [2,3,4].

The occurrence of the IAs is related to genetic, hemodynamic, and inflammatory factors, which are responsible for the bulging of the arterial wall and, ultimately, its rupture.

The precise molecular mechanisms underlying these events, however, remain unclear, although their understanding may theoretically lead to counteract this cascade.

In this study, we overview the most recent advances in the comprehension of the pathogenesis of IAs, focusing on the implications of gene mutations, inflammation pathways, and hemodynamic stress in the growth and rupture of the aneurysm. The role of neuroimaging in detecting aneurysm wall inflammation as well as that of target therapies in preventing rupture are also discussed.

## 2. Genesis of Intracranial Aneurysms

### 2.1. Genetic and Extrinsic Risk Factors

Over the last decade, several studies have delineated the structural differences between sporadic aneurysms and hereditary ones. Among these is the Familial Intracranial Aneurysm (FIA) study, a multicentric retrospective and prospective trial focused on the genome reconstruction of 475 families harboring IAs [5]. Results revealed that familial IAs is a dissimilar entity, with a higher incidence at a young age and a predilection for the middle cerebral artery (MCA). They are frequently large and multiple, and the patterns of inheritance include autosomal dominant transmission and anticipation [6,7,8,9,10].

Furthermore, many genetic syndromes were found to be susceptible to IA growth. Ehlers–Danlos syndrome type IV is a connective tissue disorder caused by the mutation of the COL3A1 gene, which transcripts for type III procollagen. The abnormal synthesis of collagen results in vascular fragility and then IA formation [11,12]. In patients affected by adult polycystic kidney disease, the risk of IAs increased by 40%, of which 25% were multiple [13,14]. The Floating-Harbor, an autosomal dominant genetic syndrome, is characterized by the SRCAP mutation. SCARP is an SNF2-related chromatin-remodeling ATPase protein, involved in endothelial integrity and repairing mechanisms [15]. Other rare hereditary diseases are strongly related to IA growth, such as neurofibromatosis type I, Marfan’s syndrome, hereditary hemorrhagic telangiectasia, and alpha-1-antitrypsin deficiency [16,17,18,19,20].

Conversely, the sporadic aneurysms occurred more frequently in females who were older than 50 years. The higher occurrence during the postmenopausal period is due to the loss of the anti-inflammatory role of estrogens. The main extrinsic risk factors for IAs are cigarette smoking, arterial hypertension, hypercholesterolemia, alcohol consumption, drug abuse, and oral contraceptives. All of these increase the oxidative stress and activation of the inflammatory cascade, resulting in chronic arterial damage [21].

In addition, abnormalities of specific chromosomal loci are linked to IA genesis. Genome-wide linkage analysis reported a higher incidence of IAs currently within the mutation of TP53, antisense inhibitor gene/chromosome 9, cyclin-dependent kinase inhibitor 2B, G572C gene of IL-6, and mutation of SOX17/chromosome 8 [22,23,24,25,26].

Particularly, the SOX17 factor is involved in physiological vascular remodeling and arterial homeostasis. SOX17-deficient rat models treated with angiotensin II (Ang-II) infusion were proven to be more susceptible to IAs formation [27]. Conversely, in 2014, Peña Silva et al. reported that the expression of angiotensin 1–7 (Ang-1–7), which binds Mas receptors and acts against the hypertensive effects of Ang-II, inhibits the aneurysmal growth. They induced IAs in wild-type and Mas receptor-deficient rats through Ang-II infusion and intracranial elastase injection. The study group was also treated with Ang-1–7. Results showed a decreased IAs formation and rupture in the study group. These data support the protective role of Ang-1–7 with a Mas receptor-dependent mechanism [28].

Moreover, the T786C polymorphism of the nitric oxide synthase (NOs) gene reduces the vasoprotection mediated by nitric oxide (NO), and the mutation of the endothelin receptor A gene leads to a dysregulation of the arterial tone. Both of these conditions significantly increase the risk of IAs [29,30].

In 2007, Pimiento and colleagues investigated the role of endothelial NOS (eNOS) in IAs formation and growth. They compared aneurysmatic diameters in wild-type and eNOS-knockout mice and determined that the eNOS expression is related to the enlargement of the IAs wall [31].

The same year, Abruzzo et al. conducted a study on rat brain circulation specimens genetically modified with NOS-2, NOS-3, and plasminogen-activator inhibitor (PAI)-1 knockout and treated with left common carotid artery ligation. After histological analysis, two IAs were found in NOS-3-knockout rats and none in NOS-2-knockout or PAI-1-knockout mice, demonstrating that only the former is a potential predisposing factor [32].

### 2.2. Wall Shear Stress, Endothelial Dysfunction, and Role of Inflammation

Among the hemodynamic mechanisms of brain blood circulation, the wall shear stress (WSS) is the result of the tangential force impressed on the arterial walls by the pulsatile blood flow, influenced by the fluid viscosity and velocity. The endothelial function is regulated by mechanoreceptors, sensitive to the decrease or increase in WSS gradient. Both low and high WSS cause functional and morphological dysregulation in endothelial cells, especially at arterial bifurcation sites, triggering the formation of aneurysms [33,34,35,36].

The so-called endothelial dysfunction, due to the hemodynamic stress, results in matrix protein degradation, the production of oxidant and vasoconstrictive agents, inflammatory activation, leukocyte rolling, and adhesion, followed by vessel remodeling [37,38,39].

The primum movens of the inflammatory cascade is the transcription of NF-κB, which induces the expression of COX2, prostaglandin E2, and proinflammatory genes [40].

The proinflammatory signals prompt the secretion of chemokines, such as the monocyte chemoattractant protein-1 (MCP-1) and vascular cell adhesion molecule-1 (VCAM-1), which recruit leukocytes in the arterial wall. Endothelial-infiltrating lymphocytes and macrophages amplify the expression of inflammatory mediators, such as matrix metalloproteinases (MMPs), interleukins, cytokines, and the tumor necrosis factor-α (TNF-α) [41,42,43].

The importance of inflammation in IAs progression is also demonstrated by experiments on animal models. In 2017, Aoki and colleagues studied the timing of NF-kB, PGE2, and prostaglandin E receptor subtype 2 (EP2) activation, and the consequent macrophage infiltration in the IAs wall. EP2 or NF-kB-knockout mice have shown less immune activation along the vessel walls and reduced IAs growth [44].

In 2014, Starke highlighted the involvement of TNF-α in IAs progression and rupture. They induced IAs in TNF-α-knockout mice subsequently treated with TNF-α inhibitor 3,6′dithiothalidomide (DTH), and results showed a decreased incidence of IAs formation [45].

Furthermore, toll-like receptors 4 (TLR4) contribute to the maintenance of immune response. In 2020, Mitsui et al. demonstrated that TLR4-knockout mice, after aneurysmal induction, have a lower incidence of aneurysmal rupture than the control littermates [46].

These data support the role of the inflammatory signaling pathway in IAs pathophysiology.

The stochastic amplification of the inflammatory cascade provokes a phenotypic switching of the vascular smooth muscle cells (VSMCs) from a contractile phenotype to a secretory one [47,48,49,50]. Modified VSMCs are spider-like shaped, split, and incapable of excreting collagen for the extracellular matrix (ECM). Proinflammatory VSMCs also upregulate the NF-κβ, IL-1β, TNF-α, and MMPs, leading to ECM transformation, proteolytic destruction, and thinning of the tunica media [51,52,53,54]. VSMCs move to the intima layer and proliferate, resulting in intimal degeneration, namely myointimal hyperplasia [55,56].

**Figure 1:**
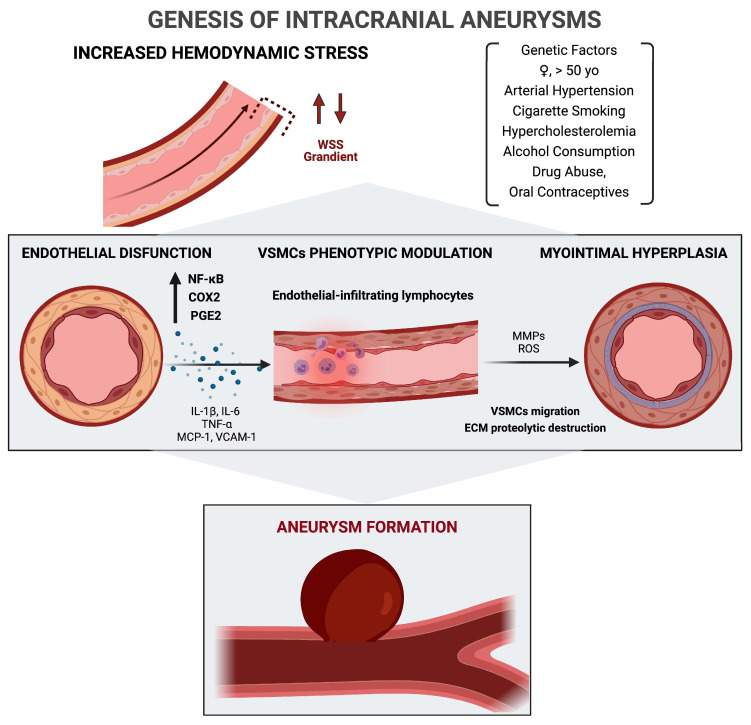
Hemodynamic and molecular mechanisms of intracranial aneurysms formation. COX2, cyclooxygenase-2; ECM, extracellular matrix; IL:, interleukin; MCP-1, monocyte chemoattractant protein-1; MMP, matrix metalloproteinase; PGE2, prostaglandin E2; ROS, reactive oxygen species; TNF-α, tumor necrosis factor-α; VCAM-1, vascular cell adhesion molecule-1; VSMC, vascular smooth muscle cell; WS, wall shear stress.

The oscillatory gradient of WSS, endothelial dysfunction, and VSMCs phenotypic modulation progressively stretch and destroy the arterial wall, contributing to IA genesis (Figure 1).

## 3. Hemodynamic Phenotypes and Patterns of Growth

**Figure 2:**
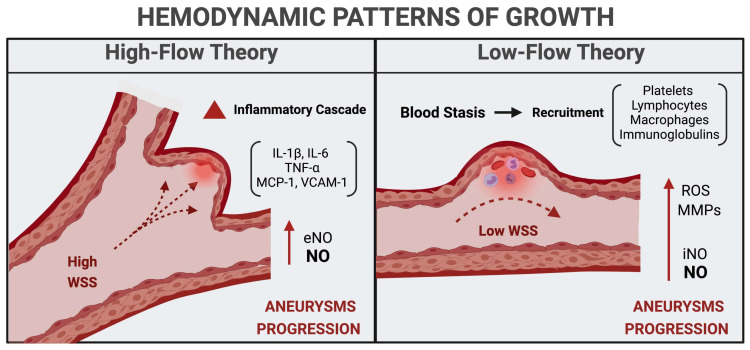
Hemodynamic patterns of intracranial aneurysms growth. eNOS, endothelial nitric oxide synthase; IL, interleukin; iNOS, inducible nitric oxide synthase; MCP-1, monocyte chemoattractant protein-1; MMP, matrix metalloproteinase; NO, nitric oxide; ROS, reactive oxygen species; TNF-α, tumor necrosis factor-α; VCAM-1, vascular cell adhesion molecule-1; WSS, wall shear stress.

Two hemodynamic models support the role of WSS in IA progression, namely the high-flow theory and low-flow one [35]. According to the high-flow theory, the abnormal increase in WSS causes focal endothelial injuries and structural changes [57]. Prolonged biomechanical stimulation stretches collagen and elastic fibers, leading to gradual dilatation and enlargement of the IA wall. Moreover, the pressure load activates the eNOS, promoting a local overproduction of NO that acts as a strong vasodilator [58,59,60]. The low-WSS theory hypothesizes the occurrence of a slowing of the blood flow in the arterial lumen, which facilitates the recruitment of platelets, lymphocytes, macrophages, and immunoglobulins [61]. The degranulation of mast cells and macrophages stimulates the inducible nitric oxide synthase (iNOS) to activate NO and induce the upregulation of MMPs and reactive oxygen species (ROS) [62,63,64]. The oxidative stress and tissue-infiltrating inflammatory mediators promote endothelial damage, VSMC phenotypic change, and thrombosis, all of them creating the assumption of cell apoptosis and rupture of the aneurysm wall [35,65,66,67] (Figure 2).

The aforementioned flow patterns lead to two different IA hemodynamic phenotypes [68]. The progressive positive WSS creates a continuous increase in transmural pressure, driving the initiation of small, thin-walled, and translucent IAs. Otherwise, the aberrant blood stagnation over-activates inflammatory pathways and atherosclerotic-like processes at the arterial wall, which result in the formation of large, thick-walled, and thrombosed IAs.

## 4. Risk of Rupture

### 4.1. Histopathological Findings

Specific histological hallmarks have been reported for the IA wall: the disruption of the tunica media and their replacement with modified non-contractile VSMCs, loss of the fibers of the internal elastic lamina, myointimal hyperplasia, thinned and shattered ECM, and proinflammatory endothelial cell dysfunction [69,70,71].

**Figure 3:**
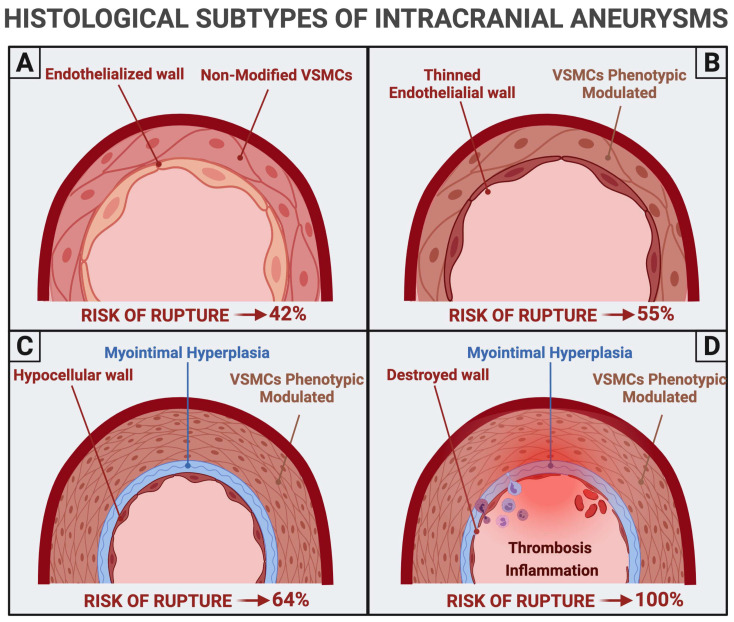
Histological subtypes of intracranial aneurysms and risk of rupture. (**A**) Type A; (**B**) type B; (**C**) type C; (**D**) type D.

In 2004, Frosen and colleagues described four aneurysmal wall histological subtypes with a progressively increased risk of rupture. Type A is marked by an endothelialized wall and non-modified VSMCs; type B harbors modified VSMCs and a thin endothelial wall; type C is characterized by the hypocellular wall, modified VSMCs, and myointimal hyperplasia; type D involves hypocellular and destroyed wall with traces of thrombosis [72] (Figure 3). The reported average risk of rupture is 42%, 55%, 64%, and 100% for type 1, type 2, type 3, and type 4, respectively [72].

### 4.2. Size, Angioarchitectural Features, and Intra-Aneurysmal Flow

In 1998, the International Study of Unruptured Intracranial Aneurysms (ISUIA) analyzed the natural history of 2621 IAs, describing a rupture rate of 0.05%/year for IAs smaller than 10 mm and 1%/year for larger ones [62]. In 2003, the prospective part of the ISUIA trial established the cut-off size at 7 mm [3]. Later, Morita et al. confirmed that the risk of rupture progressively increases based on the size, and it becomes relevant over 7 mm [73]. They also found a different likelihood of rupture according to the site, with a risk of 2%, 1.7%, 1.3%, 0.65%, and 0.2% for the basilar tip, posterior communicating, anterior communicating, MCA, and paraclinoid internal carotid artery, respectively [73].

**Figure 4:**
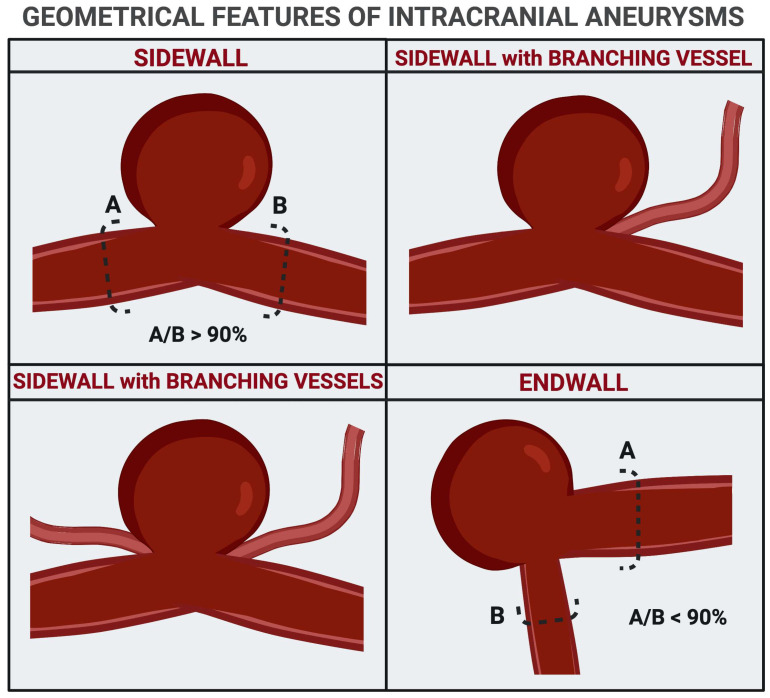
Angio-architectural classification of intracranial aneurysms.

The explanation of these data lies in the distribution of hemodynamic forces in the different locations of IAs. Based on rheologic properties and relationships with neighboring arteries, the saccular aneurysms were classified as sidewall (SWA), sidewall with branching vessels (SWBVA), and endwall (EWA) [74]. The SWAs are the focal outpouching of one artery where the percentage ratio between the pre- and post-aneurysmal segment is >90%. The SWBVAs are similar but hold smaller side branches. In EWAs, the so-called bifurcation aneurysms, the pre- and post-aneurysmal segments, have a different diameter at the neck, and their percentage ratio is <90% (Figure 4).

Angiographic computational flow techniques allows us to comprehend the intra-aneurysmal hemodynamic patterns and flow velocity in the different regions of the IA.

The low WSS proved to be responsible for the rupture of SWAs at the neck or dome [75,76,77]. Blood stasis has been identified as the major input to the atherosclerotic-like wall remodeling and formation of intraluminal thrombi via the recruitment of platelets and inflammatory mediators [76,78,79,80].

Conversely, high-WSS regions represent the breaking points for EWAs and SWBVAs. Depending on the angioarchitecture, the points of pressure impingement may mainly involve bifurcations, narrow necks, or the take-off of collateral branches [81,82].

These findings demonstrated a close relationship between the risk of rupture and angioarchitecture. The diameter of the neck, aspect ratio (dome/neck ratio), size ratio, height-width ratio, flow angle, and presence of one or more collateral branches all affect the risk [75,83,84].

In addition, age older than 60 years, female gender, hypertension, cigarette smoking, alcohol abuse, and hypercholesterolemia seem to contribute to the growth and rupture of the aneurysm [85,86,87]. In 2012, Greving et al. introduced a grading system with the aim to predict the five-year risk of rupture for IAs. The PHASES score includes patients’ demographics, clinical data, size and site of aneurysms, and the occurrence of previous bleeding. A PHASES score rating >3 is highly suggestive of rupture and provides an indication for treatment [88,89].

## 5. Future Perspectives: Imaging of Inflammation and Target Therapies

In recent decades, many efforts have been focused on novel diagnostic tools for the rupture risk stratification and treatment choice. In 2012, Hasan et al. proposed ferumoxytol-enhanced magnetic resonance imaging (FE-MRI) to predict aneurysm instability. The ferumoxytol, an intravenous iron oxide employed in anemia and renal failure, is uptaken by macrophages and lymphocytes with a peak of 24 h. The IA wall enhancement allows the estimation of the inflammatory process at the arterial wall, which reflects the active progression of aneurysmal remodeling and demolition. Therefore, the FE-MRI can predict the IA instability and improve the identification of rupture-prone aneurysms [90,91,92]. The identification of endangered IAs permits us to choose the most appropriate, tailored management.

As reported by our group, the recent advances in translational medicine, epigenetics, and immunogenomics in many neurosurgical fields have led to the proposal of new therapies targeting the inflammatory cascade [93,94,95,96,97,98,99].

The most promising approach involves the chronic use of acetylic acid (ASA) as the primary prevention for IA rupture. ASA not only irreversibly blocks the cyclooxygenase (COX)-1 and -2 but also the ROS formation, resulting in a decrease in the inflammatory cascade. In 2011, the same group of Hasan and colleagues reported the results of a comparative study on patients enrolled in the ISUIA trial and treated with ASA three times per weekly or daily. The overall data suggested a lower risk of SAH in the ASA group [100].

Statins were tested in preventing IA rupture, exploiting their ability to block upstream the inflammatory cascade. They inhibit the NF-κB activation, MMPs, and iNoS expression. In 2012, Tada and colleagues proved the dose-dependent effects of statins in the treatment of IAs. Particularly, a low dose of Pravastatin (5 mg/kg per day) was effective in avoiding the IA rupture, while a high dose of Pravastatin (50 mg/kg per day) may increase cell apoptosis and arterial wall damage [101].

COX-2 inhibitors, angiotensin receptor blockers, and mast-cell degranulation inhibitors are still under evaluation as tailored, preventive therapies to reverse the process of growth and rupture [102,103,104,105,106].

## 6. Conclusions

The hemodynamic imbalance and focal variations of the WSS were proven to be responsible for the genesis of IA.

Abnormal blood flow pulses lead to endothelial dysfunction, which causes a chronic inflammatory reaction. Leukocyte wall infiltration and the overproduction of proinflammatory mediators result in progressive remodeling of the wall, phenotypic switching of the VSMCs, myointimal hyperplasia, degradation of ECM, and aneurysmal progression.

Familial and genetic susceptibility, morphological features, and angioarchitecture significantly affect the risk of rupture.

Further advances in imaging techniques for wall instability and target therapies specifically targeted against the inflammatory pathways may significantly contribute to the improvement of the management of IA.

## Data Availability

All data are included in the main text.
